# Epimerisation of chiral hydroxylactones by short-chain dehydrogenases/reductases accounts for sex pheromone evolution in ***Nasonia***

**DOI:** 10.1038/srep34697

**Published:** 2016-10-05

**Authors:** Joachim Ruther, Åsa K. Hagström, Birgit Brandstetter, John Hofferberth, Astrid Bruckmann, Florian Semmelmann, Michaela Fink, Helena Lowack, Sabine Laberer, Oliver Niehuis, Rainer Deutzmann, Christer Löfstedt, Reinhard Sterner

**Affiliations:** 1 Institute of Zoology, University of Regensburg, 93053 Regensburg, Germany; 2 Department of Biology, Lund University, SE-22362 Lund, Sweden; 3 Department of Chemistry, Kenyon College, Gambier, OH 43022, USA; 4 Institute of Biochemistry, Genetics and Microbiology, University of Regensburg, 93053 Regensburg, Germany; 5 Institute of Biophysics and Physical Biochemistry, University of Regensburg, 93053 Regensburg, Germany; 6 Centre for Molecular Biodiversity Research, Zoological Research Museum Alexander Koenig, 53113 Bonn, Germany

## Abstract

Males of all species of the parasitic wasp genus *Nasonia* use (4*R*,5*S*)-5-hydroxy-4-decanolide (*RS*) as component of their sex pheromone while only *N. vitripennis* (*Nv*), employs additionally (4*R*,5*R*)-5-hydroxy-4-decanolide (*RR*). Three genes coding for the NAD^+^-dependent short-chain dehydrogenases/reductases (SDRs) NV10127, NV10128, and NV10129 are linked to the ability of *Nv* to produce *RR*. Here we show by assaying recombinant enzymes that SDRs from both *Nv* and *N. giraulti* (*Ng*), the latter a species with only *RS* in the pheromone, epimerise *RS* into *RR* and *vice versa* with (4*R*)-5-oxo-4-decanolide as an intermediate. *Nv*-derived SDR orthologues generally had higher epimerisation rates, which were also influenced by NAD^+^ availability. Semiquantitative protein analyses of the pheromone glands by tandem mass spectrometry revealed that NV10127 as well as NV10128 and/or NV10129 were more abundant in *Nv* compared to *Ng*. We conclude that the interplay of differential expression patterns and SDR epimerisation rates on the ancestral pheromone component *RS* accounts for the evolution of a novel pheromone phenotype in *Nv*.

Many insects rely on sex pheromones for mate location, recognition and acceptance^1^. The encoded chemical information needs to be reliable to avoid costly sexual interactions or even mismating with closely related species that may use similar chemical signals. Hence, speciation is often accompanied by a diversification of the chemical signals that enable exclusive channels for sexual communication and result in behavioural isolation of the involved species^2^,^3^. The genetic and biochemical mechanisms underlying pheromone diversification and pheromone perception, however, are only poorly understood, although some progress has been made in research on moths^4^,^5^,^6^,^7^,^8^,^9^ and fruitflies^10^,^11^,^12^,^13^. In addition to these taxa, the parasitic wasp genus *Nasonia* has become an important model system to study the molecular mechanisms underlying pheromone diversification in insects. The genus consists of four species, *N. vitripennis* (*Nv*), *N. giraulti* (*Ng*), *N. longicornis* (*Nl*) and *N. oneida* (*No*), all of which parasitize the pupae of fly species^14^,^15^,^16^. While *Nv* is a cosmopolitan species, the distribution of the other species is restricted to North America. *Nv* is sympatric with *Ng* and *No* in eastern North America and with *Nl* in the west^14^,^15^. Interspecific mating is possible although the likelihood that a female accepts a heterospecific male depends on the species combination and female’s age^17^,^18^,^19^. However, in most combinations, interspecific mating results in all male broods due to *Wolbachia*-mediated cytoplasmic incompatibility^20^. Hence, mechanisms enabling behavioural isolation to avoid costly interspecific sexual interactions of sympatric *Nasonia* species have evolved. One of these mechanisms is mate discrimination during courtship presumably mediated by species specific female cuticular hydrocarbons and male derived aphrodisiac pheromones of yet unknown chemical structure^17^,^18^,^19^,^21^. Additionally, a mechanism for mate discrimination by volatile sex pheromones has evolved in *Nv. Nasonia* males produce volatile sex pheromones to attract virgin females^21^,^22^,^23^,^24^. All species of the genus produce the two pheromone components consisting of the major component (4*R*,5*S*)-5-hydroxy-4-decanolide (*RS*) and the minor compound 4-methylquinazoline (MQ). The pheromone of *Nv*, however, contains significant amounts of a third component, (4*R*,5*R*)-5-hydroxy-4-decanolide (*RR*) (ca. 30% of the amount of *RS*)^22^,^25^. The presence of *RR* allows *Nv* females to discriminate between the pheromone of conspecific males and the less complex blend of other *Nasonia* species^18^,^25^. Quantitative trait locus (QTL) analyses using *Nv* and *Ng* as well as RNAi gene knockdown experiments revealed that an array of three very similar genes (*NV10127, NV10128* and *NV10129*), coding for putative short-chain dehydrogenases/reductases (SDRs), accounts for the pheromone difference between the two species^25^. It remained unclear, however, which of the three SDRs are actually involved and what their exact biochemical functions are.

SDRs are NAD(P)^+^/NAD(P)H-dependent oxidoreductases catalysing a variety of biochemical redox reactions^26^,^27^. Many SDRs catalyse the oxidation of hydroxyl groups or the reduction of carbonyls to alcohols, but they may also function as epimerases^26^,^27^,^28^,^29^. Therefore, it has been suggested that the *Nasonia* SDRs might synthesise *RR* by inverting the stereochemistry at carbon atom five with (4*R*)-5-oxo-4-decanolide (ODL) occurring as an intermediate^25^. It is still unknown, however, whether the *Nasonia* SDRs actually use *RS* as a substrate and, if so, whether they act alone as epimerases or concertedly by catalysing the oxidation and the reduction successively. Orthologues of the three SDR-encoding genes also exist in the genome of *Ng*, a species that, according to previous reports, does not contain *RR* in its pheromone blend. This raises the question of whether the ability of *Nv* males to synthesise *RR* is due to sequence-related differences in the catalytic activity of the SDRs or differential SDR expression. Previous SDR gene expression analyses by qPCR in abdomens of *Nv* and *Ng* revealed higher expression of NV10127 in males and of NV10129 in females^25^ but no data are available for isolated pheromone glands at the protein level.

In the present study, we expressed the SDR genes of *Nv* and *Ng* heterologously in *Escherichia coli* to study the catalytic activities of the recombinant enzymes. Specifically, we monitored the activity of the SDRs on synthetic precursors at different NAD^+^/NADH ratios. Furthermore, we performed proteomic analyses of the male pheromone glands by liquid chromatography/tandem mass spectrometry (LC-MS/MS). Finally, we conducted *in vivo* labelling experiments using ^13^C-labelled pheromone precursors to study whether the epimerisation of *RS* via the intermediate ODL occurs in live *Nasonia* males.

## Results

**Figure 1:**
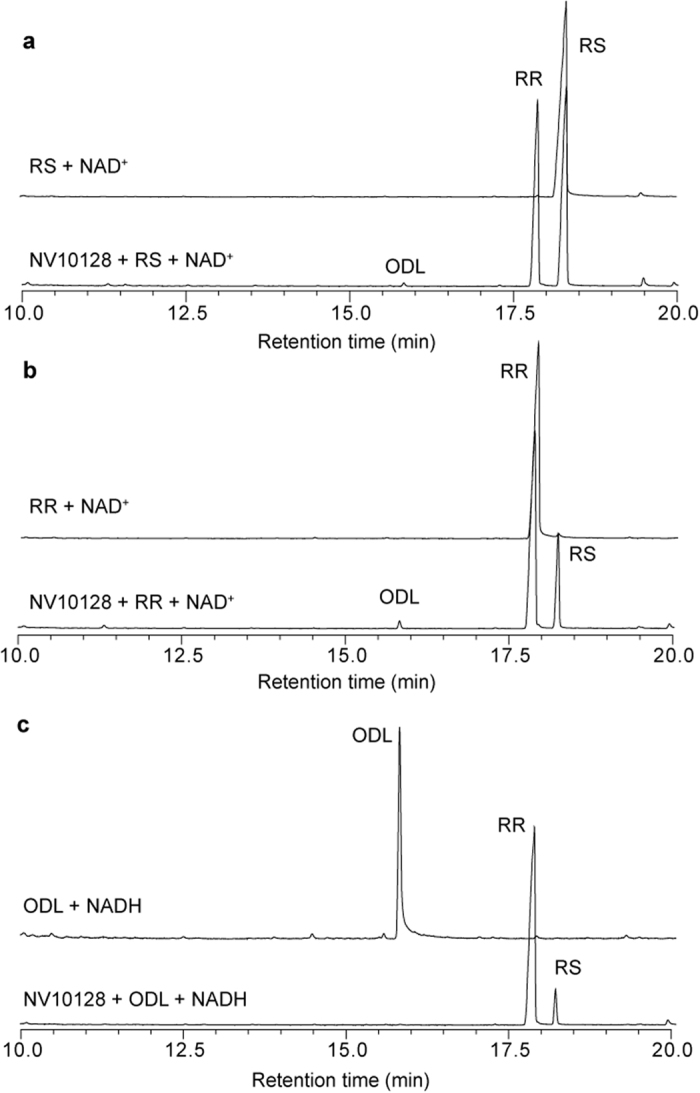
Epimerisation and reduction capacity of recombinant SDR NV10128 from *Nasonia vitripennis*. Representative total ion current chromatograms of dichloromethane extracts obtained after incubating NV10128 for 5 h with (**a**) (4*R*,5*S*)-5-hydroxy-4-decanolide (*RS*) + 1.5 mM NAD^+^, (**b**) (4*R*,5*R*)-5-hydroxy-4-decanolide (*RR*) + 1.5 mM NAD^+^, or (**c**) (4*R*)-5-oxo-4-decanolide (ODL) and 1.5 mM NADH. The upper chromatograms in each panel show the control treatments in which the protein was omitted.

**Figure 2:**
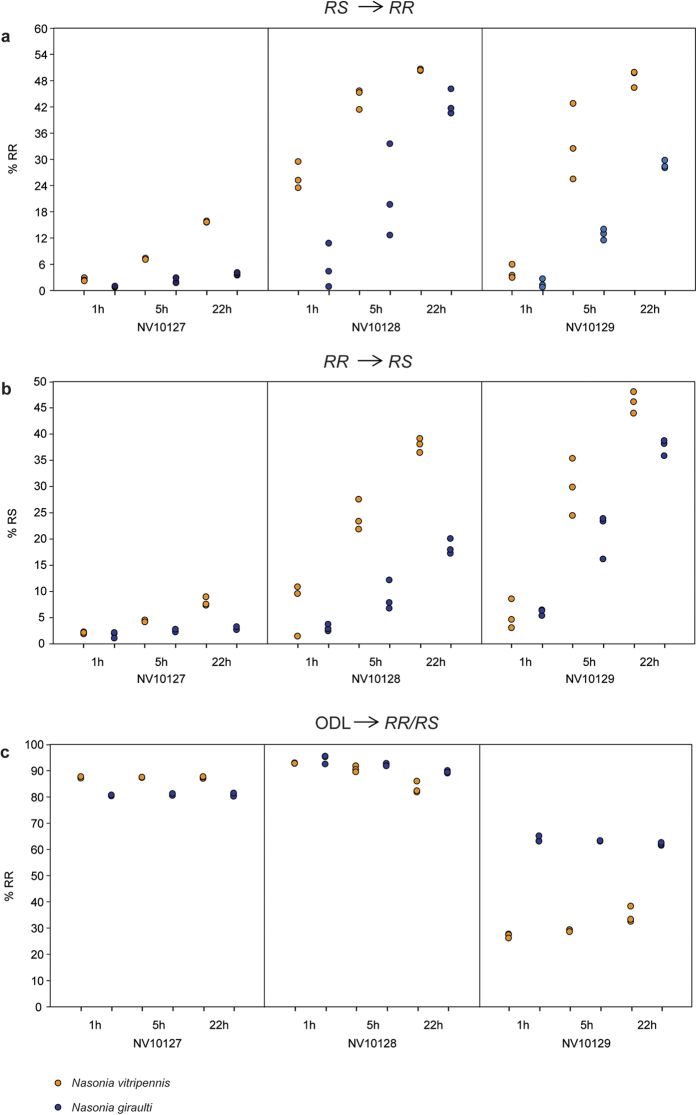
Functional characterisation of recombinant SDRs from *Nasonia* wasps. Percentage of (**a**) *RR* and (**b**) *RS* formed (individual data points from *n* = 3 replicates are given) by NV10127, NV10128 and NV10129 from *N. vitripennis* and *N. giraulti* after addition of 0.5 mM *RS* and *RR*, respectively in the presence of 1.5 mM NAD^+^ as coenzyme; (**c**) percentage of *RR* formed by the same enzymes after addition of 0.5 mM (4*R*)-5-oxo-4-decanolide (ODL) in the presence of 1.5 mM NADH as coenzyme (summed peak areas of *RS* + *RR* = 100%).

**Figure 3:**
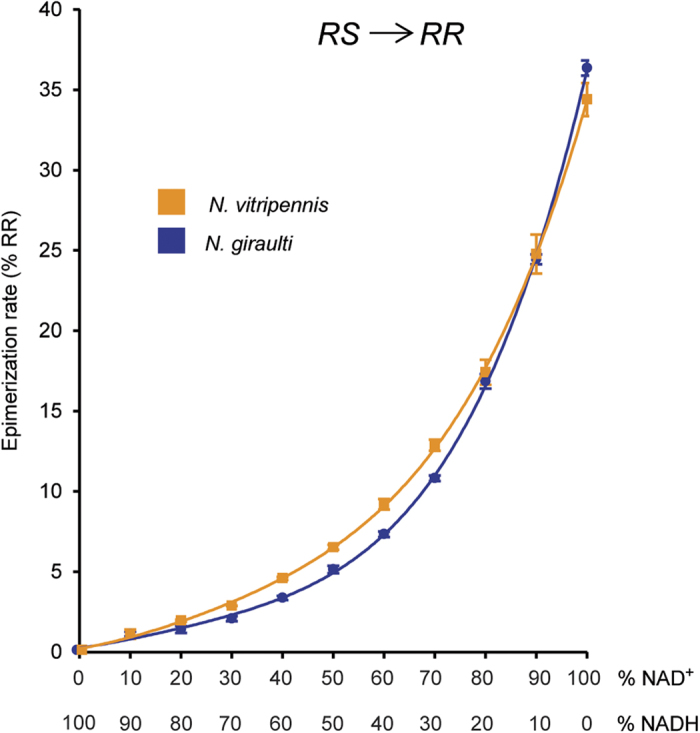
Influence of the coenzyme availability on the epimerisation rate of NV10128. Percentage of *RR* (mean + SEM, *n* = 3 for each coenzyme status) formed by NV10128 from *N. vitripennis* (orange) and *N. giraulti* (blue) after the addition of *RS* in the presence of different NAD^+^/NADH ratios (total coenzyme concentration 1.5 mM, summed peak areas of *RS* + *RR* = 100%, reaction time 5 h).

To study the catalytic activities of the SDRs under controlled experimental conditions, we introduced the coding sequence of each of the three SDRs (*NV10127, NV10128*, and *NV10129*) from *Nv* and *Ng* into an *E. coli*-based expression system. After purification of the recombinant SDRs, which do not contain any signal peptides, prosthetic groups, or metal ions, we incubated equal amounts of each enzyme for 1, 5, and 22 h with enantiopure *RS* or *RR* in the presence of NAD^+^ or an equimolar mixture of NAD^+^/NADH as coenzymes. GC/MS analysis of the reaction products revealed that all six SDRs epimerised *RS* into *RR* and *vice versa* (Fig. 1). Epimerisation rates were dependent on the coenzyme availability (Supplementary Fig. S1) and differed between the various SDRs (i.e., identity of the SDR protein and species-specific origin) (Fig. 2). Although it is plausible that these differences are due to steric constraints at the active site, it is difficult to identify the responsible amino acid residues in the absence of a high-resolution crystal structure with bound substrate and coenzyme. NV10128 and NV10129 converted *RS* and *RR* with a higher epimerisation rate than NV10127, and *Nv*-derived SDRs were generally faster than the respective orthologues from *Ng*. The epimerisation rate of *Nv*-derived NV10128 with *RS* as substrate was striking as indicated by an abundance of 26±1.8% *RR* after a reaction time of only 1 h. Therefore, we studied the effect of coenzyme availability on the epimerisation rate in more detail with NV10128 of either species by increasing the NAD^+^/NADH ratio of the reaction mixture stepwise from 0 to 100%. Epimerisation rate of NV10128 from both species increased exponentially with increasing NAD^+^ availability (Fig. 3). The presence of small amounts of ODL in the reaction mixtures of the epimerisation experiments (Fig. 1a,b) showed that ODL is likely the intermediate formed during epimerisation. To confirm this conclusion, we incubated the SDRs for 1, 5 and 22 h with synthetic ODL in the presence of NADH, the reduced form of the coenzyme which is necessary to reduce ODL into the hydroxylactones. All SDRs reduced the keto group of ODL readily to *RR* and *RS* (Fig. 1c). Less than 5% of the added ODL was detectable after 1 h in all reactions and after 5 h, all enzymes had reduced more than 99% of the ODL. However, the stereoselectivity of the reduction differed between the SDRs (Fig. 2c). While NV10127 and NV10128 of either species produced *RR* biased ratios (>80% *RR*) of HDL, NV10129 produced higher proportions of *RS* and *Nv*-derived NV10129 produced a *RS*-biased HDL ratio.

Since the sequence-related catalytic characteristics of the SDRs alone did not explain the exclusive occurrence of *RR* in *Nv*, we asked whether species-related differences in SDR expression contribute to the unique pheromone composition of *Nv* males. The pheromones of *Nasonia* males are synthesised in the rectal vesicle^30^. Pheromone biosynthesis starts after emergence of the adult males and titres maximise in 2-day-old males^22^. Therefore, we studied the functional expression of SDR genes in the pheromone glands of 1–2 d old males of either species by a proteomic approach using LC-MS/MS. To this end, protein samples derived from 10 rectal vesicles were subjected to SDS-PAGE (*n* = 3 per species). Subsequent LC-MS/MS analysis of in-gel trypsin digested proteins revealed the presence of NV10127 and NV10128/NV10129 in the rectal vesicles of *Nv* males in all three replicates as depicted in Supplementary Fig. S2. Due to the very high sequence similarity of NV10128 and NV10129^25^, these two SDRs could not be distinguished based on the peptides detected. No peptide sequence uniquely present in either of the two SDRs was found. Peptides derived from NV10127 covered a greater proportion of its sequence compared to peptides from NV10128/NV10129 (Table 1). A quantitative difference in protein expression levels is reflected by the emPAI-values (exponentially modified protein abundance index), which can be used for an approximate relative quantitation of proteins in a mixture^31^. This index suggests that NV10127 is the most abundant of the three SDRs in the rectal vesicles of *Nv* males. According to the mass spectrometric analysis, the SDRs NV10127 and NV10128/NV10129 are also expressed in rectal vesicles of *Ng* males (Supplementary Fig. S3). However, numbers of detected peptides, sequence coverage and emPAI-values are lower compared to the SDRs of *Nv*, suggesting a lower expression level in *Ng* (Table 1). Moreover, NV10128/NV10129 could only be detected in one of the three replicates with *Ng*.

Our results show that *Ng*, a species thought to produce no *RR*^25^, has the substrate *RS* and minor amounts of the SDR NV10127 and NV10128/NV10129 in the pheromone gland. This led us to hypothesise that, in contrast to previous reports^25^, at least traces of *RR* should also be detectable in *Ng*. A targeted search for *RR* in *Ng*-derived pheromone extracts revealed that traces of *RR* are indeed present (1.8 ng ± 0.2 ng/wasp (mean ± SEM), Supplementary Fig. S4).

**Figure 4:**
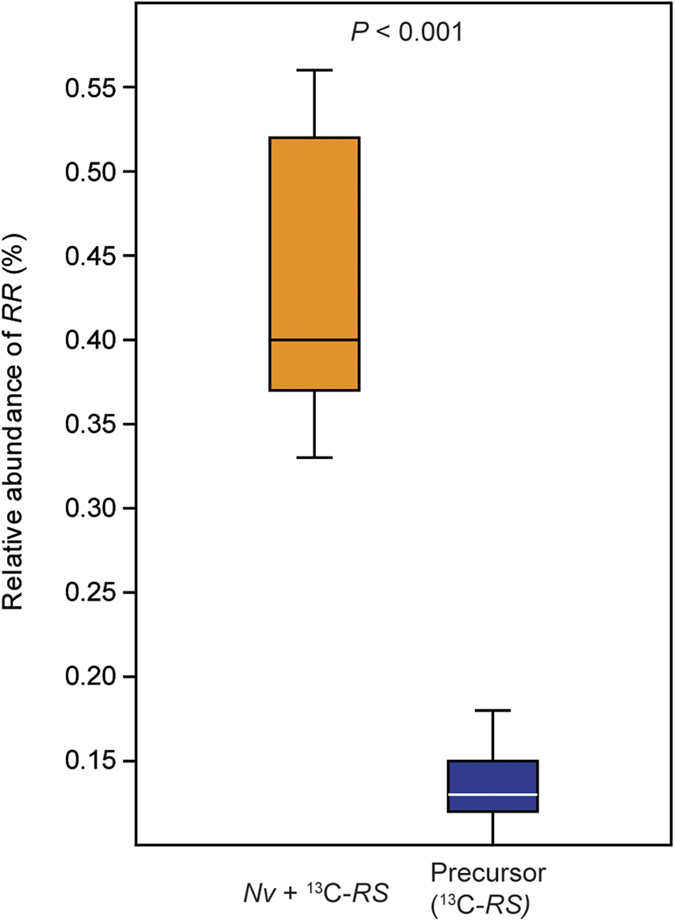
*In vivo* epimerisation of ^13^C-labelled *RS* into *RR* by *Nasonia vitripennis* males (*Nv*). Data show the relative abundance (horizontal line: median, box: 25–75 percent quartiles, whiskers: maximum/minimum range) of the diagnostic ion m/z 90 in the mass spectrum of *RR* calculated by integration of the mass trace m/z 90 at the retention times of *RR* and *RS* and relating the peak area of *RR* to the summed peak area of *RR *+ *RS*. Left column: pheromone analyses (*n* = 9) of *Nv* males (orange) treated with a purified pheromone extract containing partially ^13^C-labelled *RS* as a precursor. Right column: multiple analysis (*n* = 11) of the *Ng*-derived (blue) precursor extract to determine the amounts of ^13^C-labelled *RR* present *a priori* and to evaluate the variability of the method (statistical analysis by Mann-Whitney-*U*-test).

**Figure 5:**
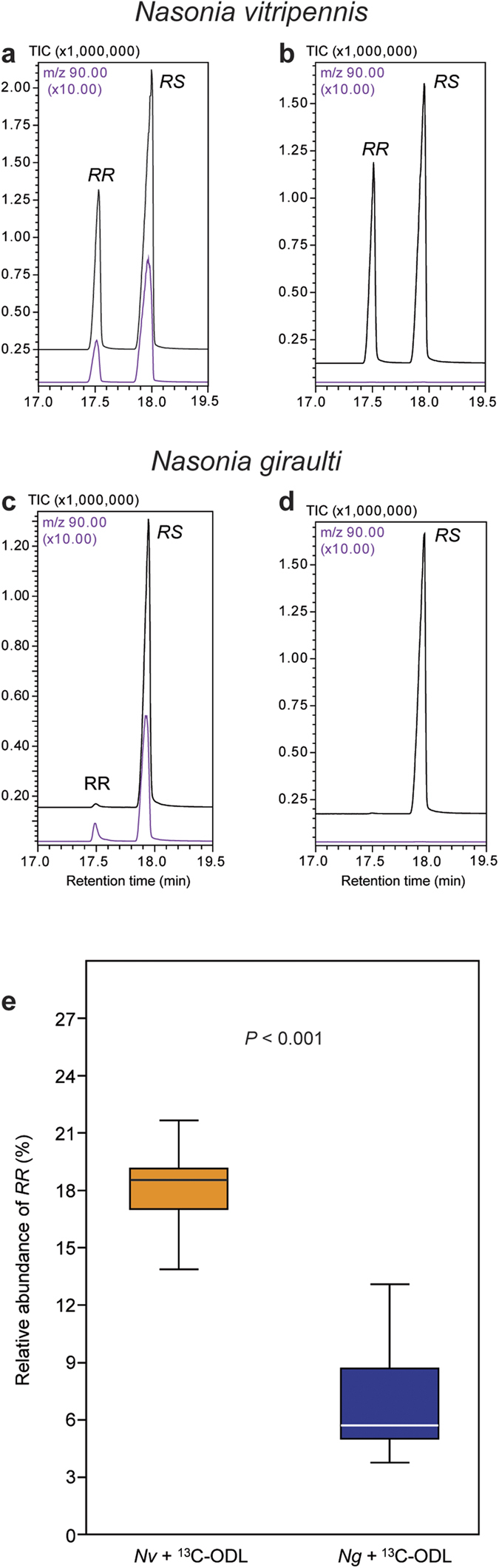
*In vivo* reduction of partially ^13^C-labelled ODL to *RR* and *RS* by living *N. vitripennis* (*Nv*) and *N. giraulti* (*Ng*) males. Shown are representative total ion chromatograms (TIC) and mass traces (diagnostic ion m/z 90, in purple) of pheromone extracts of (**a**) *Nv* and (**c**) *Ng* males treated with partially ^13^C-labelled ODL and (**b**,**d**) for control with the pure solvent. (**e**) Relative abundance (horizontal line: median, box: 25–75 percent quartiles, whiskers: maximum/minimum range) of labelled *RR* as found in the pheromone extracts (difference to 100% =*RS*, statistical analysis by Mann-Whitney-*U*-test, *n* = 10).

To demonstrate that the epimerisation of *RS* to *RR* via the formation of ODL is a process occurring in live *Nv* males, we performed ^13^C *in vivo* labelling experiments. We used *Ng* males to produce partially ^13^C-labelled *RS* and ODL. For this purpose, we reared *Ng* males on hosts that had been experimentally enriched in ^13^C linoleic acid, a known precursor of *RS* and *RR* in *Nasonia* wasps^32^. *Ng* males reared on these hosts produced *RS*, which was 64.2 ± 2.6% ^13^C-labelled (Supplementary Fig. S5). Again, the *Ng* extracts contained traces of *RR* (0.14 ± 0.06%). We used an aliquot of the labelled *RS* to synthesise ^13^C-labelled ODL (Supplementary Fig. S6). Subsequently, we applied the ^13^C-labelled *RS* or ODL to the abdomen of *Nv* males and extracted their pheromone the following day. Males treated with labelled *RS* had significantly more labelled *RR* in their pheromone than the *Ng*-derived precursor (Mann-Whitney-*U*-test, *P* < 0.001, Fig. 4). This demonstrates that *Nv* males are able to epimerise *RS* into *RR in vivo. Nv* males treated with ^13^C-labelled ODL reduced the precursor to *RS* and *RR*, with a product ratio similar to the ratio found in untreated *Nv* males (Fig. 5a–e)^22^. Surprisingly, *Ng* males also produced significant amounts of *RR* when treated with ^13^C-labelled ODL. However, this was significantly less than the amounts typically found in *Nv* (Mann-Whitney-*U*-test, *P* < 0.001; Fig. 5a–e). No residue of the applied ^13^C-labelled ODL was detectable in the extracts of either species.

## Discussion

**Figure 6:**
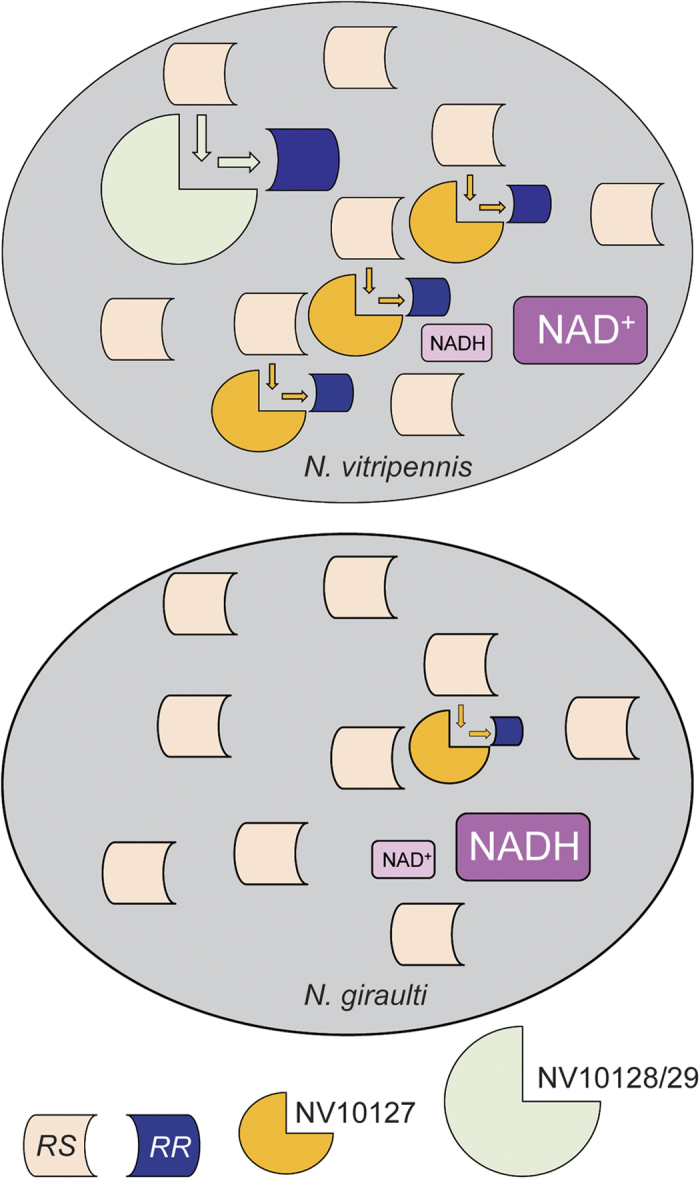
Proposed biochemical model explaining the differing pheromone composition of *N. vitripennis* (*Nv*) and *N. giraulti* (*Ng*) males. (4*R*,5*S*)-5-hydroxy-4-decanolide (*RS*) is the primary pheromone component in both species. A significant proportion (ca. 30% of the total pheromone^22^) of *RS* is epimerised in *Nv* to the diastereomer (4*R*,5*R*)-5-hydroxy-4-decanolide (*RR*) by the NAD^+^-dependent SDRs NV10127, NV10128 and/or NV10129 that are more abundant in *Nv* than in *Ng*. Additionally, the *Nv*-derived SDRs have higher epimerisation rates. In contrast, the pheromone glands of *Ng* contain only traces of *RR*. Different redox states (NAD^+^/NADH ratios) in the pheromone glands might additionally contribute to the higher abundance of *RR* in *Nv*. Differing epimerisation rate is indicated by the size and differing abundance by the number of icons.

The present investigation demonstrates that all of the studied SDRs from *Nv* and *Ng* are capable of catalysing the epimerisation of *RS* into *RR*, as well as the reverse reaction, using NAD^+^ as coenzyme with ODL as an intermediate. However, there are clear sequence-related differences in the epimerisation activity of NV10128 and NV10129 from either species compared to the less active NV10127. In general, the orthologues from *Nv* were more effective in epimerising HDL than the respective enzymes from *Ng*. Nevertheless, our assays suggest that species-specific allelic variations of the SDRs alone are not sufficient to explain the pheromone difference between *Nv* and *Ng*, because the *Ng*-derived SDRs are also capable of epimerising the hydroxylactones. However, the semiquantitative LC-MS/MS analyses of the pheromone gland proteins revealed that both NV10127 and NV10128/NV10129 were more abundant in *Nv* than in *Ng* as indicated by the much higher emPAI values (Table 1). These differences in SDR concentrations might also contribute to the species specific pheromone compositions. Our data furthermore demonstrate that the coenzyme status has a strong influence on the epimerisation efficiency of the SDRs. Thus, the differing availability of NAD^+^ of the two species might be another factor contributing to the high abundance of *RR* in *Nv* (for a summarising model explaining the differing pheromone composition of *Nv* and *Ng* males see Fig. 6). However, a differing redox status would presumably influence not only the pheromone biosynthesis but also several other cell functions. Thus, potential differences in the redox status might be restricted to particular compartments of the pheromone producing gland such as the rectal papillae where at least some steps of the pheromone biosynthesis take place^30^.

**Figure 7:**
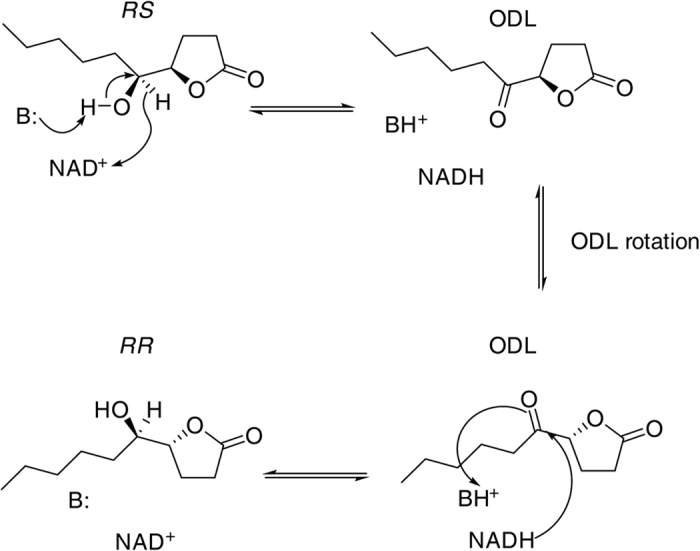
Proposed mechanism for the epimerisation of *RS* to *RR* via ODL by the *Nasonia* SDRs in analogy to the mechanism known from UDP-galactose 4-epimerase. As a first step of the epimerisation, the catalytic base (Tyr 158, B:) deprotonates the hydroxyl group and carbon 5 is concomitantly oxidised by the bound coenzyme NAD^+^ forming ODL and NADH. ODL then rotates 180°, thus presenting the opposite side of the substrate to the NADH. Subsequently, the ketone resident in ODL is reduced by hydride transfer from NADH ultimately resulting in a stereochemical inversion of the hydroxyl group.

The enzymes investigated in this study are characterised by very high amino acid sequence similarities both within and between species (Supplementary Fig. S7, for an analysis of the phylogenetic relationships of the three SDRs within and between both *Nasonia* species see Niehuis *et al*.^25^), with NV10128 and NV10129 sharing more than 97% amino acid residues while NV10127 has only 79% or 83% of the amino sequence in common with NV10128 and NV10129, respectively. The lower amino acid sequence similarity of NV10127 compared to the other two SDRs is reflected by its lower epimerisation activity. However, the activity of NV10127 to reduce ODL is comparable to that of NV10128 and NV10129. The enzymes studied here are “classical” NAD^+^/NADH dependent SDRs *sensu* Persson *et al*.^27^, thus exhibiting the typical highly conserved amino acid sequence motifs found in this class of biocatalysts (Supplementary Fig. S7). The binding site of the coenzyme is characterised by a TGxxxGxG motif and the preference for NAD^+^/NADH rather than NADP^+^/NADPH as a coenzyme is predicted by a negatively charged amino acid residue at the end of the second β-strand (here: Glu 45)^27^,^33^. The active site of most SDRs includes a S–Y–K triad (here: Ser 146, Tyr 158 and Lys 162) with an anionic Tyr acting as the catalytic base, Ser stabilising the substrate and Lys interacting with the nicotinamide ribose, thus lowering the pKa value of the Tyr-OH^27^,^34^. Mutational and structural analyses using 3β/17β-hydroxysteroid dehydrogenase as a model have revealed that a conserved Asn residue (here: Asn 115) is also involved in the catalytic mechanism^35^. SDRs are oxidoreductases with a wide range of biological functions including the functions described here, i.e., carbonyl and alcohol oxidoreduction^26^. The *Nasonia* SDRs catalyse both steps and thus function as epimerases. The best studied SDR with epimerase activity is UDP-galactose 4-epimerase (GALE), catalysing the interconversion between UDP-glucose and UDP-galactose within the Leloir pathway^28^,^29^,^36^. GALE contains a tightly bound NAD^+^ molecule, which stays attached to the enzyme during the reaction cycle and undergoes different redox state changes. After oxidation of galactose at carbon atom four and concomitant reduction of NAD^+^ to NADH, the resulting 4-ketopyranose rotates within the active site by about 180°, thus presenting the opposite side of the substrate to the NADH. Subsequently, the keto group of the sugar is reduced by hydride transfer from NADH, eventually resulting in a stereochemical inversion of the substrate’s C4 hydroxyl group^26^,^28^,^29^. We propose a similar mechanism for the stereochemical inversion at carbon atom five of the hydroxylactones catalysed by the *Nasonia* SDRs (Fig. 7). The differing epimerisation rates of the *Nasonia* SDRs might be explained by steric constraints that influence the mobility of the substrate molecule within the active site^37^,^38^. The proposed mechanism implies that most of the intermediate ODL is reduced to the epimer without leaving the enzyme-substrate complex. This and the fact that the reduction of ODL occurs very quickly (Fig. 2c) might explain why ODL has not been detected in the pheromone glands of *Nasonia* wasps so far. The application of ^13^C-labeled ODL to the abdomen of *Ng* males resulted in the formation of significant amounts of *RR* by these males while only minute amounts of *RR* occur in untreated wasps. We therefore conclude that ODL does not occur in significant amounts as a free intermediate in the pheromone glands of *Nasonia* males and that the traces of *RR* detected in *Ng* result from the epimerisation of *RS* catalysed by the diluted SDRs in the pheromone glands of *Ng* males (Table 1).

The fact that all SDR genes are also expressed in female wasps of both species^25^ suggests that the SDRs might have additional functions in *Nasonia*. Strikingly, the *Nasonia* SDR sequences have high sequence similarity with 15-hydroxyprostaglandin dehydrogenases (15-PGDH), enzymes that deactivate prostaglandins by oxidation of the 15(*S*) hydroxyl group^39^. Given the numerous hormonal functions prostaglandins have in insects^40^, the SDRs studied here might have evolved secondarily from 15-PGDHs. Characterisation of these putative ancestral functions, identification of possible alternative substrates and clarification of the structure-activity relationships underlying epimerase activity of the SDRs will help to further disentangle the mechanisms underlying sex pheromone evolution in *Nasonia*.

## Methods

### Insects

*Nv* originated from the inbred strain Phero01, which was also used in previous pheromone studies 22,24,25,30. *Ng* originated from the inbred strain NGVA2 and were kindly provided by Thomas Schmitt (University of Würzburg, Germany). Both species were reared on puparia of the green bottle fly, *Lucilia caesar*, as described elsewhere^41^. Wasps of defined age and mating status were obtained by dissecting hosts 1–2 days prior to emergence of the wasps and isolating single all-black parasitoid pupae in 1.5 ml microcentrifuge tubes until eclosion.

### Chemical syntheses

#### (4*R*,5*R*)-5-hydroxy-4-decanolide

Enantiopure *RR* was synthesised by Sharpless Asymmetric Dihydroxylation from ethyl (4*E*)-dec-4-enoate (Molekula, Gillingham, UK) as described by Garbe & Tressl^42^.

#### (4*R*,5*S*)-5-hydroxy-4-decanolide

Enantiopure *RS* was prepared from *RR* by a Mitsunobu^43^ inversion of the C5 hydroxyl group. To a solution of pure *RR* (820 mg, 4.4 mmol) in THF (80 mL) at 0 °C was added *p*-nitrobenzoic acid (1.5 g, 8.8 mmol, Acros) and triphenylphosphine (2.5 g, 9.7 mmol, Acros). The resulting mixture was stirred until the solid reagents dissolved (5 min) and a solution of diethyl azodicarboxylate (40% in toluene, 4.4 mL, 9.7 mmol, Sigma-Aldrich) was added dropwise over 5 min. The cooling bath was removed and the reaction mixture was let warm to room temperature and stirred overnight. The volatiles were removed by rotary evaporation and the residue was purified by silica gel column chromatography (eluent: 20% ethyl acetate in hexanes) to give the ester intermediate as a mixture with triphenylphosphine. This mixture was dissolved in dry methanol (20 mL) and potassium carbonate (20 mg, 0.14 mmol) was added. The reaction mixture was let stir at room temperature for 2 h, the volatiles were removed, and the residue was purified by silica gel column chromatography (eluent: 20% ethyl acetate in hexanes) to give pure *RS* (140 mg, 17%). The purity of the product was verified by enantioselective GC/MS as described elsewhere^22^. The structure of purified *RS* was determined by NMR (^1^H, ^13^C, COSY, HSQC, HMBC) and comparison with the *RR* isomer using standard and enantioselective GC/MS.

#### (4*R*)-5-oxo-4-decanolide

ODL was synthesised by oxidation of *RR* using Dess-Martin-Periodinane as described by Garbe & Tressl^42^.

### *In vivo* production of ^13^C-labelled precursors

We reared ca. 120 *Ng* males on hosts, which had been experimentally enriched in ^13^C-labelled linoleic acid as described elsewhere^32^. *RS* was extracted from two batches of 50 males each with 500 μl dichloromethane and purified by adsorption chromatography as described elsewhere^22^. Purified *RS* from one batch was dissolved in acetone and adjusted to a concentration of 10 μg/μl. After determination of the ^13^C incorporation rate into *RS* by GC/MS^32^, this solution was used for *in vivo*^13^C labelling (see below). The *RS* isolated from the second batch of male wasps was used to synthesise partially ^13^C-labelled ODL. For this purpose, the purified *RS* was dissolved in 500 μl dichloromethane and a spatula tip of Dess-Martin periodinane (Sigma-Aldrich) was added. The solution was shaken for 2 h at room temperature and excess periodinane was destroyed by washing the reaction mixture with 1 ml of 1 M sodium thiosulphate and 1 ml of saturated sodium hydrogen carbonate solution. The dichloromethane phase was removed, dried over sodium sulphate and analysed by GC/MS to determine the purity of ODL and the percentage of ^13^C incorporation. Finally, the solvent was removed under a stream of nitrogen and the ODL was re-dissolved in acetone (10 μg/μl) for *in vivo*^13^C labelling.

### Gene Cloning

The coding-sequences of the target genes^25^ were amplified from cDNA-containing plasmid constructs (synthesised by GeneArt AG, Regensburg, Germany) using the oligonucleotide PCR primers specified in Table 2. Amplicons were inserted into the expression plasmid pET28a(+) using the introduced *Bam*HI/*Xho*I restriction sites.

### Protein Expression and Purification

For expression of the SDR genes, *E. coli* BL21 CodonPlus (DE3) RIPL (Stratagene) was transformed with pET28a(+)-*NV10127*, pET28a(+)-*NV10128*, pET28a(+)-*NV10129* (either from *Nv* or *Ng*), respectively. Transformed cells were grown at 37 °C in lysogenic broth (LB) with 50 μg/ml kanamycin and 30 μg/ml chloramphenicol to OD_600_ = 0.5. Gene expression was induced by addition of 0.5 mM IPTG. After growth overnight at 20 °C, cells were harvested by centrifugation and disrupted by ultrasonication. The recombinant proteins carrying an N-terminal His6-tag, a thrombin cleavage site and a 14 amino acid long linker were purified from the soluble fraction of the cell extract by Ni^2+^-affinity chromatography using a His Spin Trap column (GE Healthcare). For this purpose, proteins dissolved in a dilute imidazole buffer (100 mM potassium phosphate, pH 7.5, 300 mM potassium chloride and 10 mM imidazole) were loaded onto the column and subsequently eluted with a concentrated imidazole buffer (100 mM potassium phosphate, pH 7.5, 300 mM potassium chloride and 1 M imidazole). Elution fractions containing pure protein (>95%) as determined by SDS-PAGE were pooled, dissolved in 100 mM potassium phosphate, pH 7.5 using a NAP^TM^-5 column (GE Healthcare), dropped into liquid nitrogen, and stored at −80 °C. Protein concentrations were determined by measuring the absorbance at 280 nm using molar and specific extinctions coefficients (ε_280nm_ for Nv/Ng are NV10127: 23045/23170 M^−1^ cm^−1^; NV10128: 20525/19035 M^−1^ cm^−1^; NV10129: 20525/20525 M^−1^ cm^−1^) that were calculated from the amino acid sequence (http://web.expasy.org/protparam/).

### *In vitro* epimerisation and reduction assay with purified SDRs

For the assays with recombinant SDRs, a solution of 125 μg of each protein dissolved in 1.8 ml Tris-HCl buffer (pH 7.0) was added with 100 μl of either NAD^+^, NADH or NAD^+^ + NADH (dissolved in 1 M Tris-HCl buffer, total concentration of the coenzymes: 1.5 mM) and 19 μl of either *RR*, *RS* or ODL (dissolved in ethanol). The final concentration of the precursors was 0.5 mM which is below the concentration found in the pheromone gland (ca. 0.3 M, assuming a gland diameter of 100 μm, a spherical structure of the gland and a mean *RS*/*RR* amount of 240 ng/gland^30^. When ODL was used as a precursor (reduction assay), solely NADH was employed as coenzyme. When *RR* or *RS* was used as precursor (epimerisation assay) either NAD^+^ or the 1:1 mixture of NAD^+^/NADH was employed as coenzyme to investigate whether the coenzyme availability influences the epimerisation rate of the enzymes. Additionally, we investigated the coenzyme availability in more detail by incubating *Nv*-derived NV10128 for 5 h with *RS* in the presence of varying NAD^+^/NADH ratios (0–100% NAD^+^ increased by 10% steps, total coenzyme concentration 1.5 mM).

All reactions were kept at 30 °C, shaken at a rate 300 rpm, and samples of 500 μl each were taken after 1 h, 5 h and 22 h. All samples were extracted twice in 250 μl dichloromethane and analysed by GC/MS. Each combination of protein/precursor/coenzyme/time was tested in triplicate (*n* = 3), resulting in a total of 336 assays. Control assays were conducted for each treatment as described above without adding the SDRs.

### GC/MS analysis

Chemical analyses were performed using the conditions and instrumentation described elsewhere^32^. For the detection of incorporated ^13^C in insect-derived HDL and ODL, the mass spectra at the expected retention times of *RR*, *RS* and ODL were scrutinised for the appearance of diagnostic ions (HDL: m/z 90, 107, 120, 196 Supplementary Fig. S5; ODL: m/z 46, 76, 89, 105, 194, Supplementary Fig. S6). Incorporation rates of the *in vivo* produced precursors were calculated by relating the peak areas of labelled diagnostic ions (HDL: m/z 90; ODL: m/z 105) to the total peak area of the respective labelled plus unlabelled (HDL: m/z 86; ODL: m/z 99) ions. To increase the sensitivity of the method and to detect minute amounts of ^13^C-labelled *RR/RS* in the *in vivo* labelling experiments, pheromone extracts were analysed in the selective ion monitoring (SIM) mode focusing on the diagnostic ion pairs m/z 86/90, 101/107, 115/120, and 186/196 (Supplementary Fig. S5). *In vivo* epimerisation of ^13^C-labelled *RS* into *RR* by *Nv* males was calculated by integration of the diagnostic ion trace at m/z 90 and relating the peak area of *RR* to the summed peak areas of *RR* and *RS*. These data were compared with the relative abundance of *RR* in the ^13^C-labelled precursor solution. *In vivo* reduction of ^13^C-labelled ODL to *RR* and *RS* by *Nv* and *Ng* was likewise monitored by the diagnostic ion trace at m/z 90. For analysis of the epimerisation assays with the recombinant SDRs, we related the peak area of the respective epimerisation product to the summed peak areas of the unreacted precursor and the product (*RR* + *RS* = 100%). For analysis of the reduction assay with ODL as precursor, we related the peak area of *RR* to the total peak area of both products (*RR* + *RS* = 100%).

### Protein analysis by LC-MS/MS

To isolated rectal vesicles from 10 males of either *N. vitripennis* or *N. giraulti* (n = 3 replicates per species), 40 μl NuPAGE^®^ LDS sample buffer (Invitrogen) containing 50 mM DTT were added. Samples were incubated for 20 min at 70 °C with two times vortexing in-between. After briefly spinning down cellular debris, the supernatant was subjected to SDS-PAGE on a precast 10% Bis-Tris gel (Invitrogen). Proteins were visualised by Coomassie-staining using SimplyBlue™ SafeStain (Invitrogen). For proteomic analysis, a gel lane was cut into 20 slices. The gel slices were washed consecutively with 50 mM NH_4_HCO_3_, 50 mM NH_4_HCO_3_/acetonitrile (3/1) and 50 mM NH_4_HCO_3_/acetonitrile (1/1), shrunk by adding 100% acetonitrile and lyophilised. Cysteines were blocked by reduction with DTT for 30 min at 57 °C followed by an alkylation step with iodoacetamide for 30 min at RT in the dark. Gel slices were washed again and lyophilised as described above. Subsequently, proteins were in gel-digested with trypsin (Trypsin Gold, mass spectrometry grade, Promega) overnight at 37 °C. Approximately 2 μg trypsin in 50 mM NH_4_HCO_3_ was used per 100 μl gel volume. Peptides were eluted twice with 100 mM NH_4_HCO_3_ followed by an additional extraction with 50 mM NH_4_HCO_3_ in 50% acetonitrile. Prior to LC-MS/MS analysis combined eluates were lyophilised and reconstituted in 20 μl of 1% formic acid. Peptides were separated on an UltiMate 3000 RSLCnano System (Thermo Scientific, Dreieich, Germany) by reversed-phase chromatography using a preconcentration column (C18 Acclaim Pepmap 100, 100 μm i.d. x 20 mm, Thermo Fisher) followed by a Reprosil-Pur Basic C18 nano column (75 μm i.d. x 250 mm, Dr. Maisch GmbH, Ammerbuch, Germany) in a linear gradient of 4% to 40% acetonitrile in 0.1% formic acid for 60 min at 300 nl/min. The LC-system was coupled to a maXis plus UHR-QTOF System (Bruker Daltonics, Bremen, Germany) via a CaptiveSpray nanoflow electrospray source (Bruker Daltonics). The mass spectrometer was operated in DDA mode at resolution of minimum 60000 for MS and MS/MS scans. MS/MS spectra were acquired with collision induced dissociation (CID) fragmentation. Compass 1.7 acquisition and processing software (Bruker Daltonics) allowed the use of a dynamic method with a fixed cycle time of 3 s and a m/z dependent collision energy adjustment between 34 and 55 eV. The scan rate of MS spectra acquisition was 2 Hz, the mass range of the precursor scan was set from m/z 175 to m/z 2000.

Raw data were processed in Data Analysis 4.2 (Bruker Daltonics) and processed by the Mascot database search engine using Protein Scape 3.1.3 (Bruker Daltonics). Mascot 2.5.1 (Matrix Science) was used to search the NCBI nr protein database. Furthermore, customised databases were searched comprising *N. vitripennis* and *N. giraulti* entries from NCBI supplemented with the three *N. giraulti* SDRs that were derived from cDNA sequencing and alignment^25^. Search parameters were as follows: enzyme specificity trypsin with 1 missed cleavage allowed, precursor tolerance 0.02 Da, MS/MS tolerance 0.04 Da, carbamidomethylation or propionamide modification of cysteine, oxidation of methionine, deamidation of asparagine and glutamine were set as variable modifications. The Protein Extractor function of ProteinScape facilitated protein list compilation. Finally, MS/MS spectra of *Nasonia* spp. peptides were subjected to manual validation.

### *In vivo*^13^C labelling experiments

1–2-d-old *Nv* males were isolated in microcentrifuge tubes and cold-sedated on an ice bath. Subsequently, 0.1 μl of the acetone solutions containing partially ^13^C-labelled *RS* (*n* = 9) and ODL (*n* = 10), respectively, were applied to the abdominal tip of the wasps using a 5 μl microsyringe designed for GC on-column injection (Hamilton, Bonaduz, Switzerland). The possible reduction of ODL to HDL was also tested with *Ng* males (*n* = 10). Control wasps (*n* = 10 for each species) were treated with pure acetone. After 20 h, wasps were frozen at −20 °C and extracted for 30 min with 10 μl dichloromethane. These extracts were used for GC/MS analysis. Additionally, we analysed the ^13^C-*RS* solution that was used (ten times) to quantify the minute amounts of ^13^C-labelled *RR* present in the extract before application to the wasps and to evaluate the variability of the method.

### Statistical analysis

The relative abundance of ^13^C-labelled *RR* in the *in vivo* labelling experiment and the *RR/RS* ratios resulting from the *in vivo* reduction of ODL by *Nv* and *Ng* males were compared by a two-sided Mann-Whitney-*U*-test using Past 3.0 scientific software.

## Additional Information

**How to cite this article**: Ruther, J. *et al*. Epimerisation of chiral hydroxylactones by short-chain dehydrogenases/reductases accounts for sex pheromone evolution in *Nasonia*. *Sci. Rep*. **6**, 34697; doi: 10.1038/srep34697 (2016).

## Supplementary Material

Supplementary Information

## Figures and Tables

**Table 1:** Results of the mass spectrometric analysis of SDRs in the rectal vesicles of male *N. vitripennis* (*Nv*) and *N. giraulti* (*Ng*).

Protein name	Species	Mascot score			Sequence coverage (%)			Number of peptides			Unique peptides			emPAI value^a^		
		I	II	III	I	II	III	I	II	III	I	II	III	I	II	III
NV10127	*Nv*	935.4	673.9	794.6	63.2	56.4	66.5	16	12	15	13	11	11	5.34	2.79	4.16
	*Ng*	39.3	148.0	25.5	7.1	16.5	4.5	2	4	1	1	3	1	0.23	0.51	0.11
NV10128/NV10129	*Nv*	191.6	256.8	199.6	15.9	19.7	18.9	4	4	5	2	2	1	0.51	0.51	0.67
	*Ng*	0	120.4	0	0	20.5	0	0	5	0	0	3	0	0	0.67	0

Roman numbers indicate the individual replicates. Ten rectal vesicles of 1–2 day old wasps were pooled per replicate and subjected to SDS-PAGE. In-gel trypsin digested proteins were analysed by LC-MS/MS. Protein database searching of the resulting mass spectra was performed using the Mascot search engine.

^a^exponentially modified protein abundance index.

**Table 2:** cDNA-containing plasmid constructs and oligonucleotide primers used to PCR-amplify the nucleotide sequences of SDR-encoding target genes.

Gene	Species	Plasmid	Forward and reverse oligonucleotide primer (5′ → 3′)
*NV10127*	*Nv*	13ABUYWP_LOC100113909_Nv_CDS_pMA-T	CCC GGA TCC ATG ACT AAA ATC CCA CGT GA
			CCC CTC GAG TTA CCA CAA ACT TTT TAA ATC TA
*NV10127*	*Ng*	13ABUYVP_LOC100113909_Ng_CDS_pMA-T	CCC GGA TCC ATG ACT AAA ATC TCA CG
			CCC CTC GAG TTA CCA CAA ACA TTT TAA ATC TT
*NV10128*	*Nv*	13ABUYUP_LOC100121889_Nv_CDS_pMA-T	CCC GGA TCC ATG ACT GAA ATC TCA CGT GA
			CCC CTC GAG TCA AAT GTA ATC ATG GTA ATC T
*NV10128*	*Ng*	13ABUYTP_LOC100121889_Ng_CDS_pMA-T	CCC GGA TCC ATG ACT GAA ATC TCA CGT GA
			CCC CTC GAG TCA AAT GTA ATC ATT GTA ATC TT
*NV10129*	*Nv*	13ABUYSP_LOC100121923_Nv_CDS_pMA	CCC GGA TCC ATG ACT GAA ATC TCA CGT GA
			CCC CTC GAG TCA AAT GTA ATC ATT GTA ATC TT
*NV10129*	*Ng*	13ABUYRP_LOC100121923_Ng_CDS_pMA-T	CCC GGA TCC ATG ACT GAA ATC CCA AGT GA
			CCC CTC GAG TCA AAT GTA ATC ATT GTA ATC TT
