# Supplementary material for: Epimerisation of chiral hydroxylactones by short-chain dehydrogenases/reductases accounts for sex pheromone evolution in Nasonia
Source: Sci Rep. 2016 Oct 5;6:34697. doi: 10.1038/srep34697 (PMC5050451; doi:10.1038/srep34697)
Supplement: Supplementary Information [file srep34697-s1.pdf]

Supplementary Information for "Epimerisation of chiral hydroxylactones by short-chain dehydrogenases/reductases accounts for sex pheromone evolution in *Nasonia*"

Joachim Ruther[1], Åsa K. Hagström[2], Birgit Brandstetter[1], John Hofferberth[3], Astrid Bruckmann[4], Florian Semmelmann[5], Michaela Fink[1], Helena Lowack[1], Sabine Laberer[5], Oliver Niehuis[6], Rainer Deutzmann[4], Christer Löfstedt[2] & Reinhard Sterner[5]

[1] Institute of Zoology, University of Regensburg, 93053 Regensburg, Germany.

[2] Department of Biology, Lund University, SE-22362 Lund, Sweden

[3] Department of Chemistry, Kenyon College, Gambier, OH 43022, USA

[4] Institute of Biochemistry, Genetics and Microbiology, University of Regensburg, 93053 Regensburg, Germany

[5] Institute of Biophysics and Physical Biochemistry, University of Regensburg, 93053 Regensburg, Germany

[6] Centre for Molecular Biodiversity Research, Zoological Research Museum Alexander Koenig, 53113 Bonn, Germany

* Correspondence: Email: Joachim.ruther@ur.de

Supplementary Figure S1

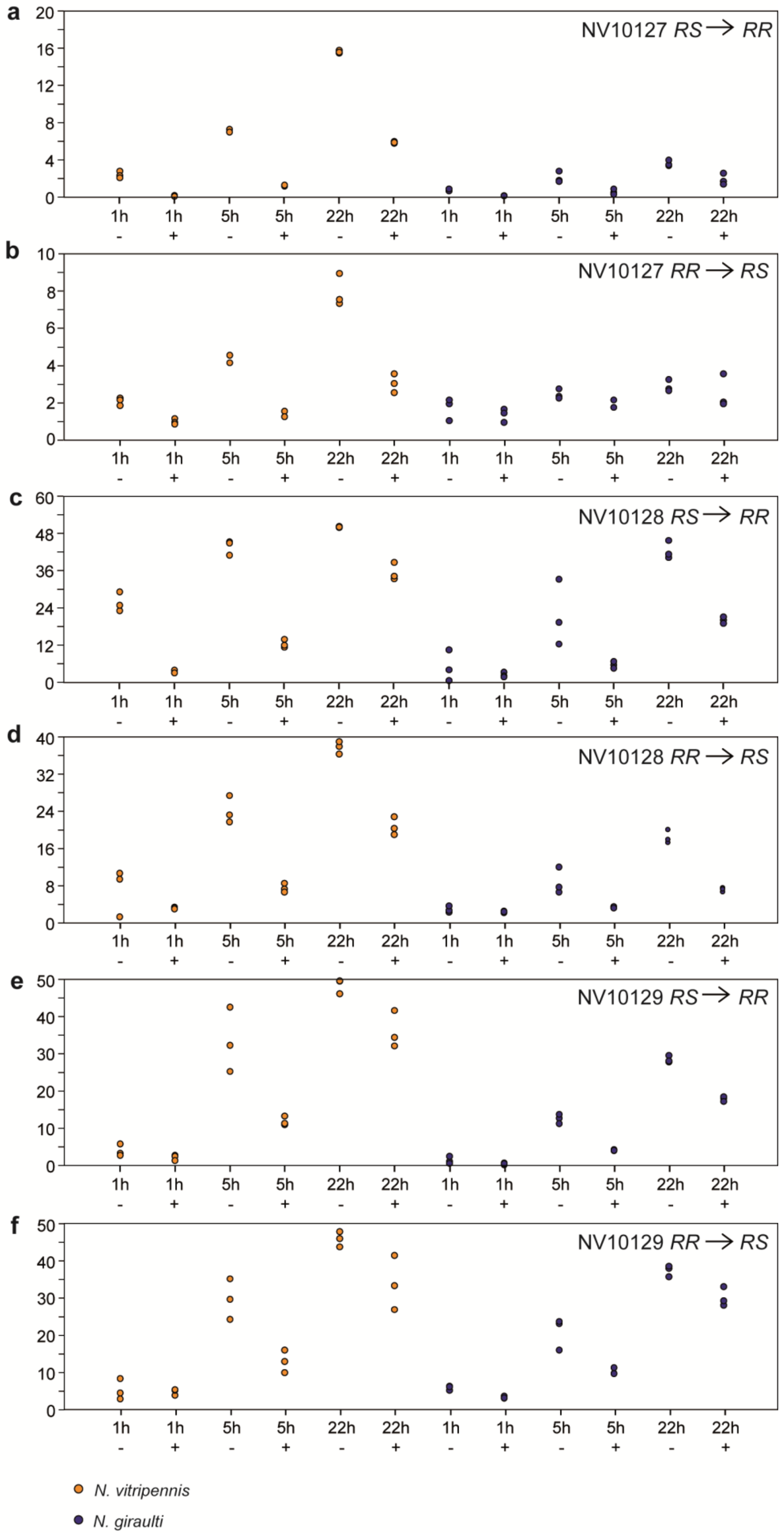

Supplementary Figure S1. Impact of coenzyme availability on the epimerisation rates of recombinant SDRs from *Nasonia* wasps. Percentage of (**a**, **c**, **e**) *RR* and (**b**, **d**, **f**) *RS* (individual data from *n* = 3 replicates are given) formed by (**a**, **b**) NV10127, (**c**, **d**) NV10128 and (**e**, **f**) NV10129 from *N. vitripennis* and *N. giraulti* after addition of 0.5 mM *RS* and *RR*, respectively, in the presence of either 1.5 mM NAD^+ alone or additionally 1.5 mM NAD^+/NADH as coenzymes. The presence/absence of NADH is indicated by "+" and "-", respectively.

Nasonia vitripennis

Replicate I

NV10127

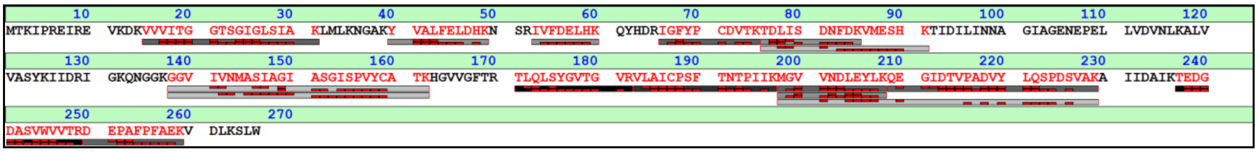

NV10128

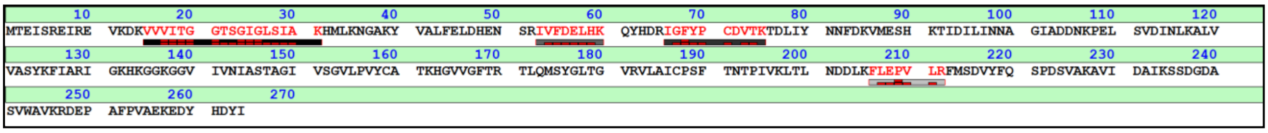

Replicate II

NV10127

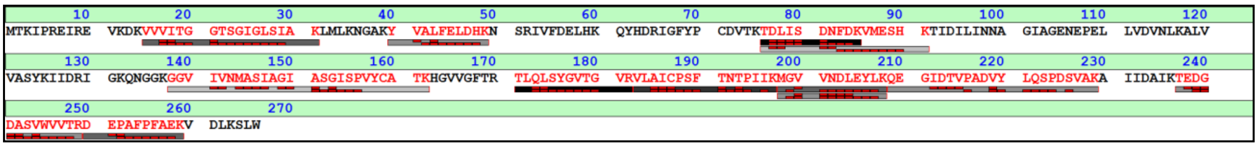

NV10128

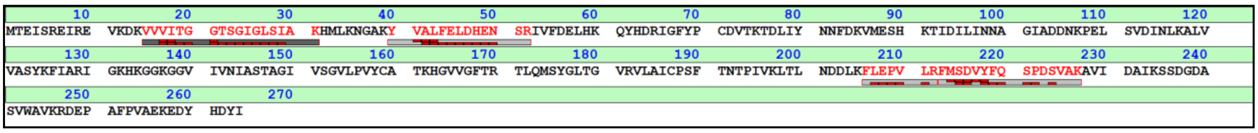

Replicate III

NV10127

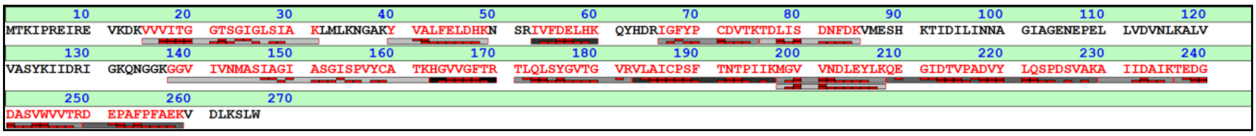

NV10128

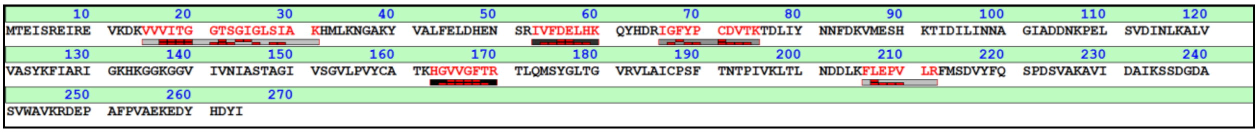

Supplementary Figure S2. Results of the mass spectrometric analysis of SDRs in the rectal vesicles of male *N. vitripennis*. Peptides detected by LC-MS/MS in each replicate are indicated in red. Please note that results are shown from analysing NV10128, although the detected peptides did not allow distinguishing between NV10128 and NV10129.

Nasonia giraulti

Replicate I

NV10127

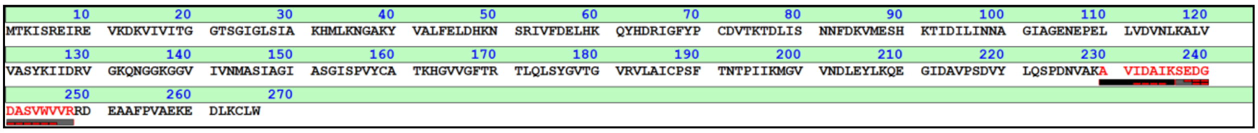

NV10128/NV10129 not detected

Replicate II

NV10127

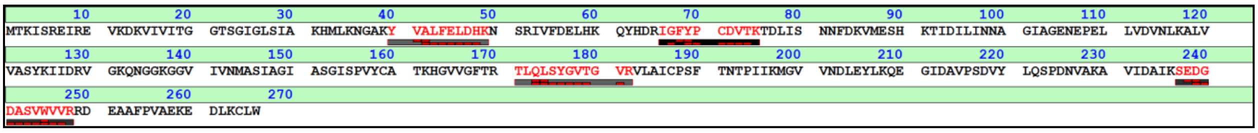

NV10128

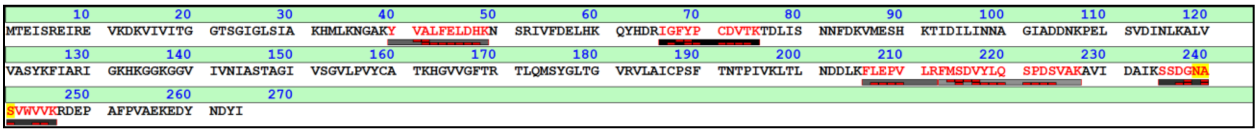

Replicate III

NV10127

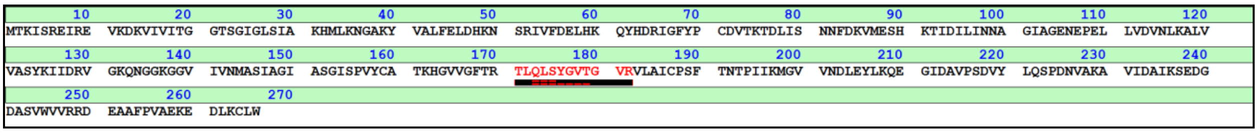

NV10128/NV10129 not detected

Supplementary Figure S3. Results of the mass spectrometric analysis of SDRs in the rectal vesicles of male *N. vitripennis*. Peptides detected by LC-MS/MS in each replicate are indicated in red. Please note that results are only shown from analyzing NV10128, although the detected peptides did not allow distinguishing between NV10128 and NV10129.

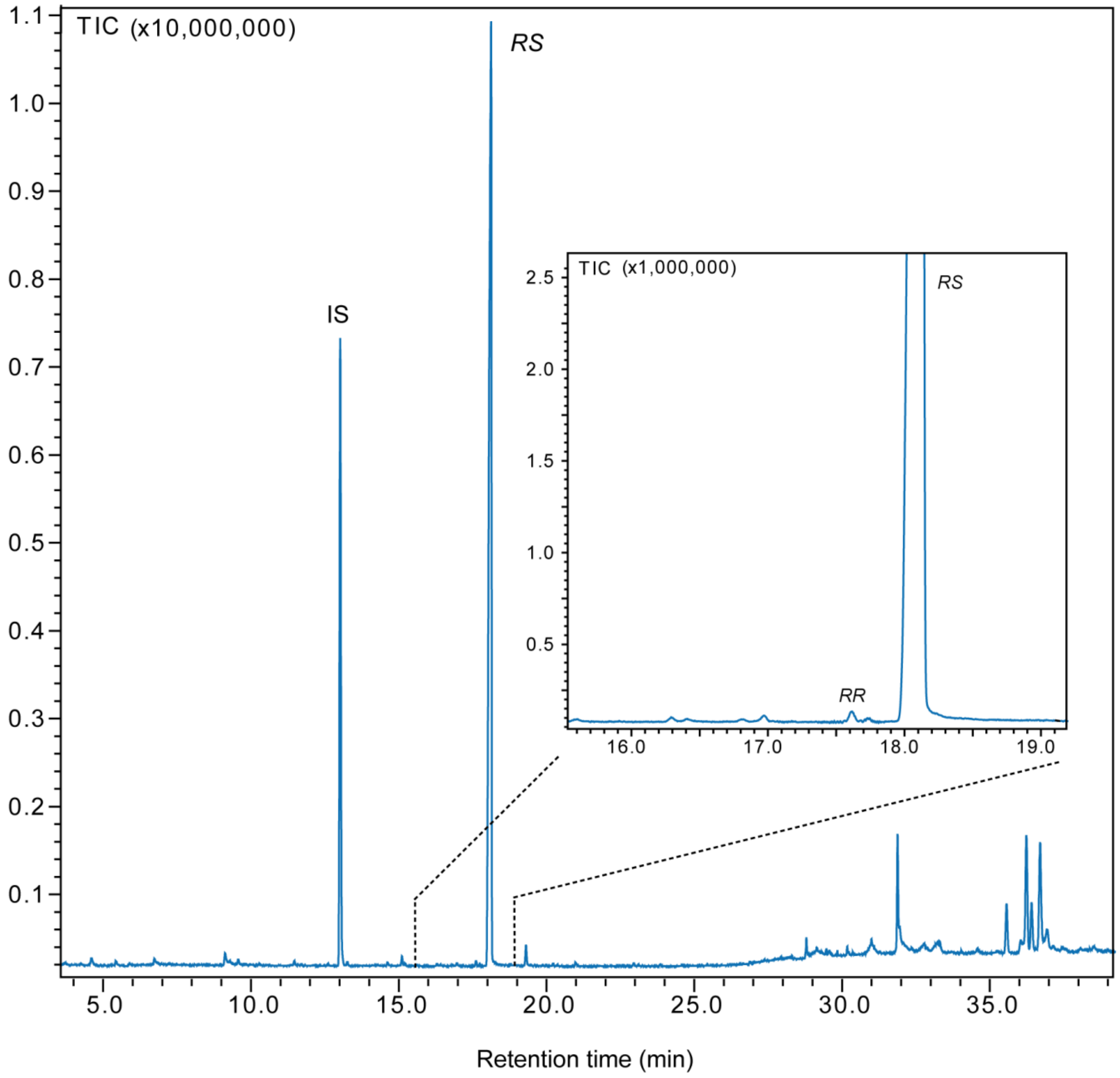

Supplementary Figure S4. Detection of trace amounts of *RR* in *Nasonia giraulti* males. Representative total ion chromatogram (TIC) of a whole body extract of a *N. giraulti* male. Males were extracted with 25 µl dichloromethane containing 10 ng/µl methyl undecanoate as an internal standard (IS). Insert shows an enlarged cut-out of the elution range of the pheromone; *RR* = (4*R*,5*R*)-5-hydroxy-4-decanolide, *RS* = (4*R*,5*S*)-5-hydroxy-4-decanolide.

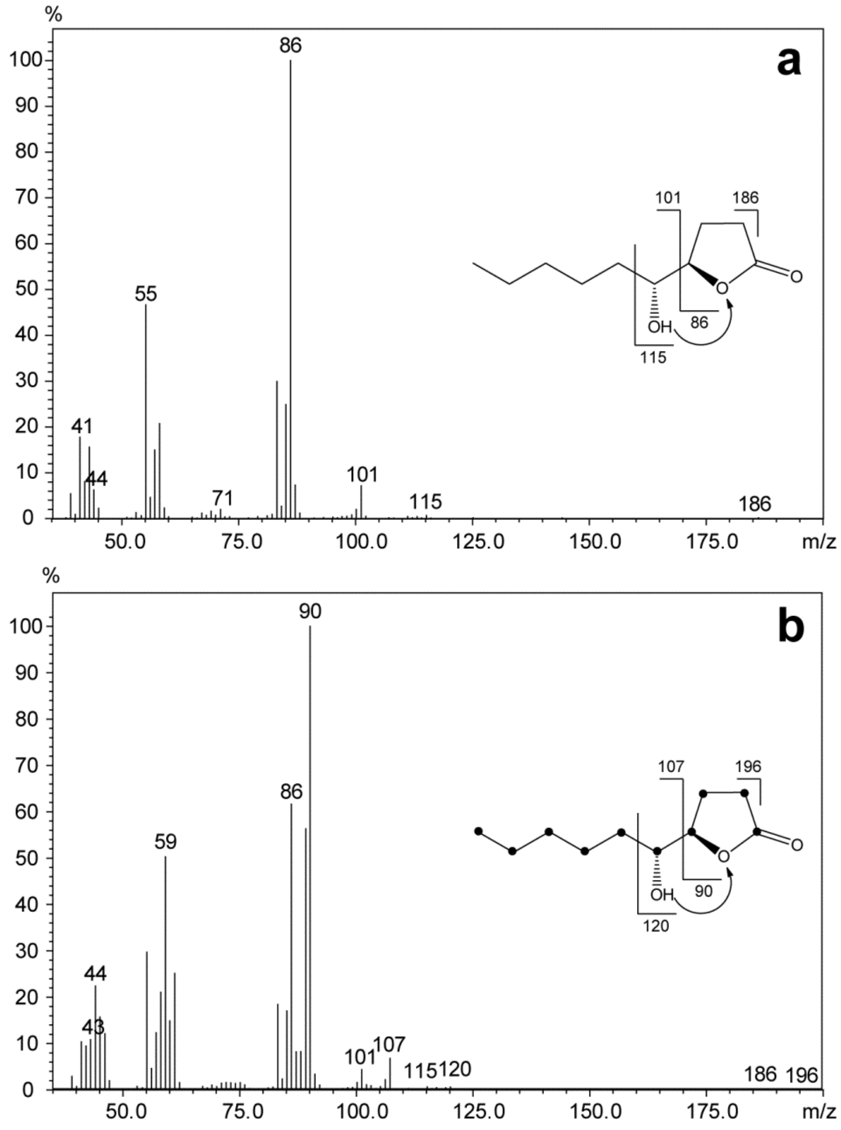

Supplementary Figure S5. Mass spectra of (a) synthetic unlabelled (4*R*,5*S*)-5-hydroxy-4-decanolide (*RS*) and (b) partially ^{13}C-labelled *RS*. The labelled compound was produced *in vivo* by *Nasonia giraulti* males grown on fly hosts enriched in ^{13}C-labelled linoleic acid. Diagnostic mass fragments are given, black dots indicate ^{13}C-atoms.

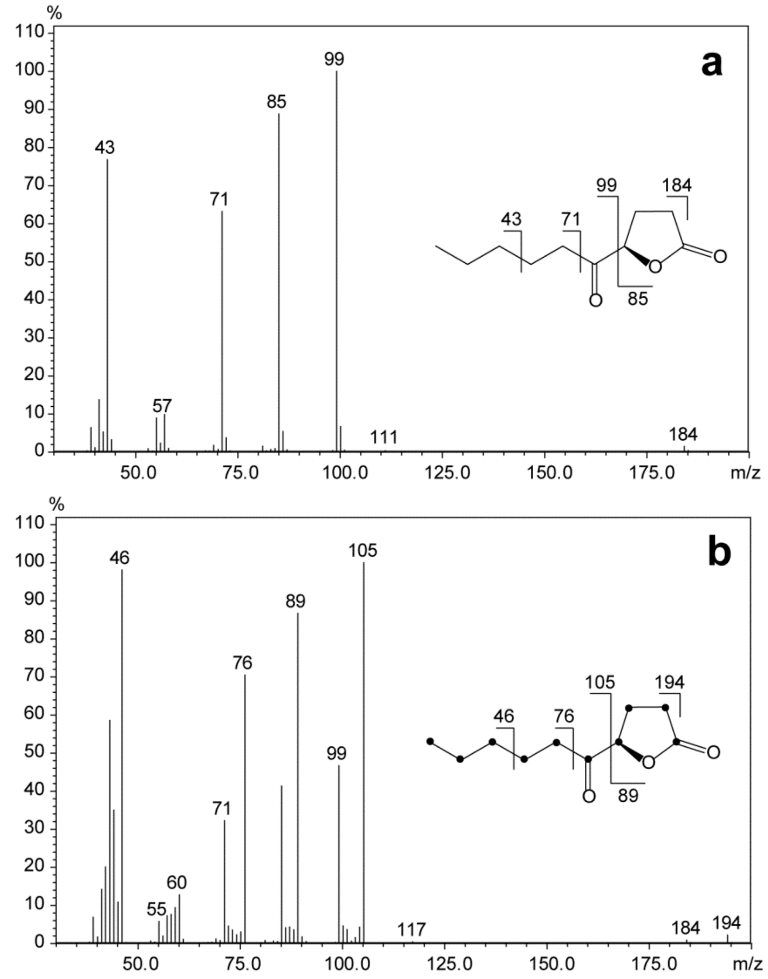

Supplementary Figure S6. Mass spectra of (a) synthetic unlabelled (4*R*)-5-oxo-4-decanolide (ODL) and (b) partially ^{13}C-labelled ODL. ODL was synthesized by Dess-Martin oxidation of partially ^{13}C-labelled (4*R*,5*S*)-5-hydroxy-4-decanolide (*RS*) from *Nasonia giraulti* males grown on fly hosts enriched in ^{13}C-labelled linoleic acid. Diagnostic mass fragments are given, black dots indicate ^{13}C-atoms.

```
10127NV   MTKIPREIREVKDKVVVITGGTSGIGLSIAKLMLKNGAKYVALFELDHKNSRIVFDELHK
10127NG   MTKISREIREVKDKVIVITGGTSGIGLSIAKHMLKNGAKYVALFELDHKNSRIVFDELHK
10128NV   MTEISREIREVKDKVVVITGGTSGIGLSIAKHMLKNGAKYVALFELDHENSRIVFDELHK
10128NG   MTEISREIREVKDKVIVITGGTSGIGLSIAKHMLKNGAKYVALFELDHKNSRIVFDELHK
10129NV   MTEISREIREVKDKVVVITGGTSGIGLSIAKHMLKNGAKYVALFELDHENSRIVFDELHK
10129NG   MTEIPSEIREVKDKVIVITGGTSGIGLSIAKLMLKNGAKYVALFELDHKNSRIVFDELHK
          **:*. *********:*************** ****************:***********

10127NV   QYHDRIGFYPCDVTKTDLISDNFDKVMESHKTIDILINNAGIAGENEPELLVDVNLKALV
10127NG   QYHDRIGFYPCDVTKTDLISNNFDKVMESHKTIDILINNAGIAGENEPELLVDVNLKALV
10128NV   QYHDRIGFYPCDVTKTDLIYNNFDKVMESHKTIDILINNAGIADDNKPELSVDINLKALV
10128NG   QYHDRIGFYPCDVTKTDLISNNFDKVMESHKTIDILINNAGIADDNKPELSVDINLKALV
10129NV   QYHDRIGFYPCDVTKTDLIYNNFDKVMESHKTIDILINNAGIADDNKPELSVDINLKALV
10129NG   QYHDRIGFYPCDVTKTDLISNNFDKVMESHKTIDILINNAGIADDNKPELSVDINLKALV
          ******************* :**********************.:*:*** **:******

10127NV   VASYKIIDRIGKQNGGKGGVIVNMASIAGIASGISPVYCATKHGVVGFTRTLQLSYGVTG
10127NG   VASYKIIDRVGKQNGGKGGVIVNMASIAGIASGISPVYCATKHGVVGFTRTLQLSYGVTG
10128NV   VASYKFIARIGKHKGGKGGVIVNIASTAGIVSGVLPVYCATKHGVVGFTRTLQMSYGLTG
10128NG   VASYKFIARIGKHKGGKGGVIVNIASTAGIVSGVLPVYCATKHGVVGFTRTLQMSYGLTG
10129NV   VASYKFIARIGKHKGGKGGVIVNIASIAGIVSGFLPVYCATKHGVVGFTRTLQMSYGLTG
10129NG   VASYKFIARIGKHKGGKGGVIVNIASIAGIVSGFLPVYCATKHGVVGFTRTLQMSYGLTG
          *****:* *:**::*********:** ***.**. ******************:***:**

10127NV   VRVLAICPSFTNTPIIKMGVVNDLEYLKQEGIDTVPADVYLQSPDSVAKAIIDAIKTEDG
10127NG   VRVLAICPSFTNTPIIKMGVVNDLEYLKQEGIDAVPSDVYLQSPDNVAKAVIDAIKSEDG
10128NV   VRVLAICPSFTNTPIVKLTLNDDLKFL--EPVLRFMSDVYFQSPDSVAKAVIDAIKSSDG
10128NG   VRVLAICPSFTNTPIVKLTLNDDLKFL--EPVLRFMSDVYLQSPDSVAKAVIDAIKSSDG
10129NV   VRVLAICPSFTNTPIVKLTLNDDLKFL--EPVLRFMSDVYFQSPDSVAKAVIDAIKSSDG
10129NG   VRVLAICPSFTNTPIVKLTLNDDLKFL--EPVLRFMSDVYFQSPDSVAKAVIDAIKSSDG
          ***************:*: : :**::*  * :  . :***:****.****:*****:.**

10127NV   DASVWVVTRDEPAFPFAEKVDLKSLW
10127NG   DASVWVVRRDEAAFPVAEKEDLKCLW
10128NV   DASVWAVKRDEPAFPVAEKEDYHDYI
10128NG   NASVWVVKRDEPAFPVAEKEDYNDYI
10129NV   DASVWAVKRDEPAFPIAEKEDYNDYI
10129NG   NASVWVVKRDEPAFPVAEKEDYNDYI
           :****.* ***.***.*** *  :
```

co-enzyme binding site[1]

acidic residue indicative of co-enzyme preference for NAD(H)[1]

adenine ring binding of coenzyme[1]

stabilization of central β-sheet[2]

substrate binding and active site[3]

critical residues of active site[1-2]

H-bonding to carboxamide of nicotinamide ring[2]

Supplementary Figure S7. Amino acid multiple sequence alignment of the SDRs from *N. vitripennis* (NV) and *N. giraulti* (NG) investigated in this study. Conserved motifs and functions are highlighted by different colours.

References

(1) Persson, B., Kallberg, Y., Oppermann, U. & Jornvall, H. Coenzyme-based functional assignments of short-chain dehydrogenases/reductases (SDRs). *Chem. Biol. Interact.* **143**, 271-278 (2003).

(2) Oppermann, U. *et al.* Short-chain dehydrogenases/reductases (SDR): the 2002 update. *Chem. Biol. Interact.* **143**, 247-253 (2003)

(3) Hoffmann, F. & Maser, E. Carbonyl reductases and pluripotent hydroxysteroid dehydrogenases of the shortchain dehydrogenase/reductase superfamily. *Drug Metabol. Rev.* **39**, 87-144 (2007).
